# Bone Loss Prevention of Bisphosphonates in Patients with Inflammatory Bowel Disease: A Systematic Review and Meta-Analysis

**DOI:** 10.1155/2017/2736547

**Published:** 2017-08-21

**Authors:** Yan Hu, Xiaoting Chen, Xiaojing Chen, Shuang Zhang, Tianyan Jiang, Jing Chang, Yanhong Gao

**Affiliations:** Department of Geriatrics, Xinhua Hospital of Shanghai Jiaotong University, School of Medicine, Shanghai 2000092, China

## Abstract

**Objective:**

The purpose of this study was to evaluate the effect of bisphosphonates in improving bone mineral density (BMD) and decreasing the occurrence rate of fractures and adverse events in patients with inflammatory bowel disease (IBD).

**Methods:**

Randomized controlled trials (RCTs) which use bisphosphonates in IBD patients were identified in PubMed, MEDLINE database, EMBASE database, Web of Knowledge, and the Cochrane Databases between 1990 and June 2016. People received bisphosphonate or placebos with a follow-up of at least one year were also considered. STATA 12.0 software was used for the meta-analysis.

**Results:**

Eleven randomized clinical trials were included in the meta-analysis. The data indicated that the percentage change in the increased BMD in the bisphosphonates groups was superior to that of the control groups at the lumbar spine and total hip. At the femoral neck, there was no significant difference between the two groups. The incidence of new fractures during follow-up showed significant reduction. The adverse event analysis revealed no significant difference between the two groups.

**Conclusion:**

Our results demonstrate that bisphosphonates therapy has an effect on bone loss in patients with IBD but show no evident efficiency at increasing the incidence of adverse events.

## 1. Introduction

Inflammatory bowel disease (IBD) includes Crohn's disease (CD), ulcerative colitis (UC), and indeterminate colitis and is characterized by chronic relapses and remitting inflammatory disorders of the gastrointestinal tract. Severe gastrointestinal symptoms including fatigue, abdominal pain, diarrhea, gastrointestinal bleeding, and damage to the structure and function of the gastrointestinal tract can occur due to this inflammation. The disease induces multisystem disorders, especially in the musculoskeletal system.

The disease itself, or chronic inflammation, smoking, glucocorticoid therapy, and so on, can cause skeletal system implications and bone disease [1, 2]. In recent years, the risk of low bone mineral density (BMD) and osteoporosis has increased in IBD patients at a prevalence between 22% and 77% compared with normal cases [3, 4]. The risk of fracture has likewise increased [5], and the risk of hip fracture has grown approximately 60% in patients with IBD [6]. The most relevant affecting factors are that patients always receive long-term corticosteroid therapy [7, 8]. Glucocorticoids increase the expression of the receptor activator for nuclear factor-kappa B ligand (RANKL) and decrease the expression of osteoprotegerin (OPG). Both of them play an important role in osteoclastogenesis to prolong the lifespan of osteoclasts [9]. Other factors such as nutritional interventions including dietary calcium and vitamin D intake and absorption, genetic factors, malabsorption, hypogonadism, bowel resection, and inflammatory cytokines such as TNF, IL-1beta, IL-2, and IL-17 may also have its impacts [10, 11].

Bisphosphonates are among the drugs most often used in the treatment of osteoporosis or osteopenia. They can be classified into two groups: nonnitrogen-containing bisphosphonates (e.g., etidronate and clodronate) and nitrogen-containing bisphosphonates (e.g., pamidronate, alendronate, risedronate, ibandronate, and zoledronate) [12]. Bisphosphonates have been found to prevent bone loss in patient with osteoporosis and corticosteroid-induced osteoporosis [13–15]. Alendronate has reported increased bone density in patients with glucocorticoid therapy. Cochrane systematic reviews have proved that alendronate and risedronate resulted in clinically important and statistically significant reductions in vertebral, nonvertebral, and hip fractures as a secondary form of prevention in postmenopausal women [16, 17]. The benefits of etidronate have also been demonstrated in the secondary prevention of vertebral fractures [17]. Moreover, calcium supplementation and vitamin D offer some benefits and somewhat prevent the development of osteoporosis or osteomalacia. Hormone replacement therapy (HRT), selective estrogen receptor modulators (SERMs), and sodium fluoride also have their effects [18, 19].

The number of people currently with IBD is increasing, and many have the potential to develop a bone disease. Focusing on early awareness in these individuals and defining the risk of fracture and adverse events in each patient to treat excessive bone loss and prevent osteoporotic fractures are important actions. According to the current limited data, bisphosphonates are the optimal choice for the therapy of both primary and glucocorticoid-induced osteoporosis in patients with IBD [20]. Several clinical trials have been conducted to analyse the effect of bisphosphonate on improving bone mineral density and decreasing fracture rates [21]. However, the effects of bisphosphonate used in patients with IBD have varied, and so have the experimental results [22]. In this meta-analysis, we unite and reanalyse previous clinical trials with the aim of developing further knowledge on the effect of bisphosphonates on bone loss and decreasing adverse events in patients with IBD.

## 2. Methods

### 2.1. Search Strategy

A literature search was performed from PubMed (1990–June 2016), MEDLINE database (1990–June 2016), EMBASE database (1990–June 2016), Web of Knowledge, and the Cochrane Controlled Trials Register in the Cochrane library. In the search, we used the terms: (inflammatory bowel disease OR ulcerative colitis OR Crohn's disease) and (osteoporosis or osteopenia or (bone and (density or mass or loss))) and (exp Diphosphonates/or (bisphosphonate^*∗*^ or alendron^*∗*^ or fosamax or etidron^*∗*^ or didronel or risedron^*∗*^ or actonel or ibandron^*∗*^ or bonivaor zolendron^*∗*^ or zometa or zomera or aclasta or reclastor pamidron^*∗*^ or aredia)). In addition, other relevant articles in hand were also searched. The language was restricted in English and the species was limited to humans in the search.

### 2.2. Study Selection

We included studies if they are randomized controlled trials (RCTs) which discussed the use of bisphosphonates in inflammatory bowel disease (IBD) patients, including the ones that only have Crohn's disease or only have ulcerative colitis. These recipients may be given calcium alone or vitamin D and calcium, while the control group received no treatment or placebo and may be given calcium alone or vitamin D and calcium. In addition, other inclusion criteria were as follows: (1) the participants' mean age being older than 18 years old; (2) the length of treatment being more than one year (including one year); (3) bisphosphonate in any dosage. There was no restriction about the gender of the participants. Duplications were excluded. BMDs were determined by dual-energy X-ray absorptiometry (DXA).

### 2.3. Statistical Analysis

In this meta-analysis, data analysis was conducted by the change in BMD values separately in the lumbar spine, total hip, and femoral neck. And the BMD values were expressed in percent change for both bisphosphonate and control groups. We used Stata/SE 12.0 program (Stata Corporation, College Station, TX, USA) for the total statistical analysis. Weighted mean differences (WMD) were calculated according to the percent change in BMD. Funnel plot was also drawn to assess the possible publication bias. The *χ*^2^ test and *I*^2^ statistic (represents the percentage of variability because of between-study variability) were applied to assess the heterogeneity among studies. If *χ*^2^  *p* value < 0.1 or *I* > 50%, the statistic heterogeneity between studies was significant. Heterogeneity among studies was considered to be statistically significant when the *p* value was less than 0.1.

Moreover, subgroup analysis on the basis of treatment duration of studies (12 months and 24 months) was conducted for a further comparison. The incidence of new fractures and adverse events (AEs) was also conducted by using odds ratio (OR) between the bisphosphonate and control group.

## 3. Results

### 3.1. Literature Selection

**Figure 1:**
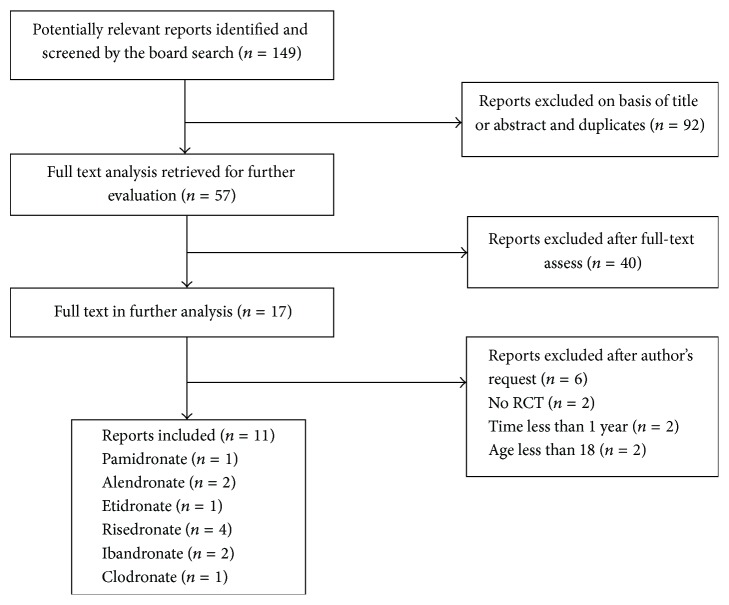
Flowchart of study selection in the meta-analysis.

According to the aforementioned search strategy and terms, 149 potentially relevant reports were found. Eleven random controlled trials (RCTs) [20, 22–31] met the inclusion criteria from these reports and others were found by additional articles that were considered eligible for this meta-analysis (Figure 1). The trials involved 785 participants. Three reports focused on participants with IBD [20, 26, 28], seven involved participants with Crohn's disease [22–25, 27, 29, 30], and one trial targeted patients with ulcerative colitis. All of the participants had osteopenia or osteoporosis. Only postmenopausal women were included in Palomba's [26] trial and excluded from Abitbol's [20] trial. Of all the eleven trials, two trials included adult patients with prolonged GC use [20, 31]. At baseline, there was generally a mild difference in the number of participants allocated to the intervention and control group except for von Tirpitz et al. [25]. There were three groups (two interventions and one placebo) in von Tirpitz et al. [25] and Klaus et al. [22]. Six studies [20, 23, 24, 26, 28, 31] were conducted in 12 months and the leaving papers were administered over the course of more than 1 year.

The characteristics of all of the trials are summarized in Table 1, including the number of patients, gender ratio, age, BMD, and duration.  Table 2 compares the number of adverse events, nonvertebral and vertebral fractures between the two groups.

### 3.2. Effect of Bisphosphonates in Lumbar Spine BMD

**Figure 2:**
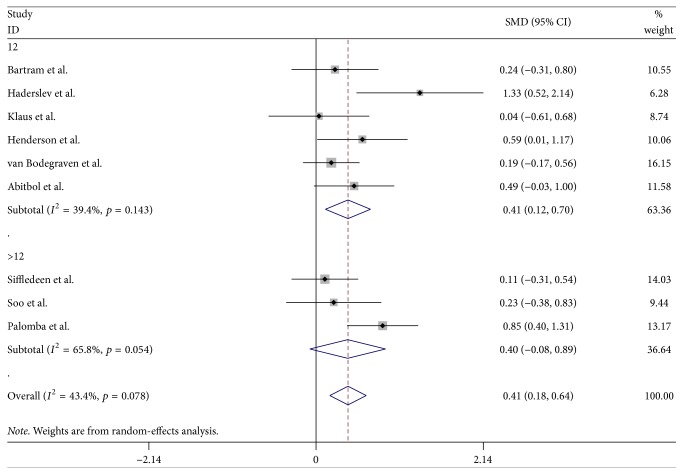
Randomized controlled trials of bisphosphonates in improving spinal BMD in IBD. Forest plot showing the weighted mean differences and 95% confidence interval of the percentage change in BMD in the lumbar spine in the bisphosphonate and control groups. A subgroup analysis of the treatment duration of the two groups is also shown.

**Figure 3:**
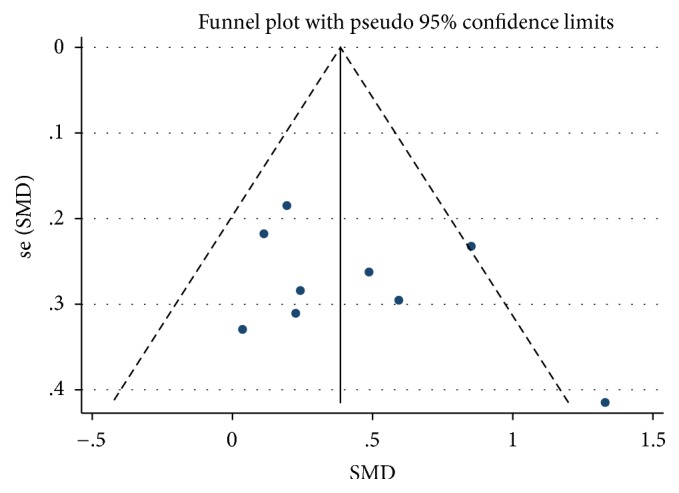
Funnel plot of studies included in Figure 2. Dots represent the results of each study. Funnel plots showing some asymmetry of nine trials reporting the efficacy of the bisphosphonates versus control groups in change of BMD in the lumbar spine in IBD patients.

Nine trials reported the percentage change of BMD in the lumbar spine in the bisphosphonate and control groups of IBD patients [20, 22–24, 26–30]. Tirpitz et al. [25] showed Δ*T* scores of spine BMD and found nonsignificant between groups. There was an increment of spine BMD values in the bisphosphonate group compared with the control group (WMD = 0.41, 95% CI: 0.18–0.64, *p* = 0.001) (Figure 2). The results of a fixed-effects model in the group were exactly the same as those of a random-effects model. Statistical moderate heterogeneity was found in this analysis (*I*^2^ = 43.4%, *p* = 0.078). Moreover, the funnel plot also revealed some asymmetry (Figure 3) and Begg's and Egger's tests ruled out a trend toward publication bias (*p* = 0.466 and 0.281, resp.). Subgroup analysis targeting on treatment duration was established to determine this heterogeneity. For 12 months, the bisphosphonate group generated an increment in BMD values, and these trials were relatively homogeneous (*I*^2^ = 39.4%, *p* = 0.143). The heterogeneity test for 24 months indicated that the statistical heterogeneity was large (*I*^2^ = 65.8%, *p* = 0.054) (Figure 2).

### 3.3. Effect of Bisphosphonates in Total Hip BMD

**Figure 4:**
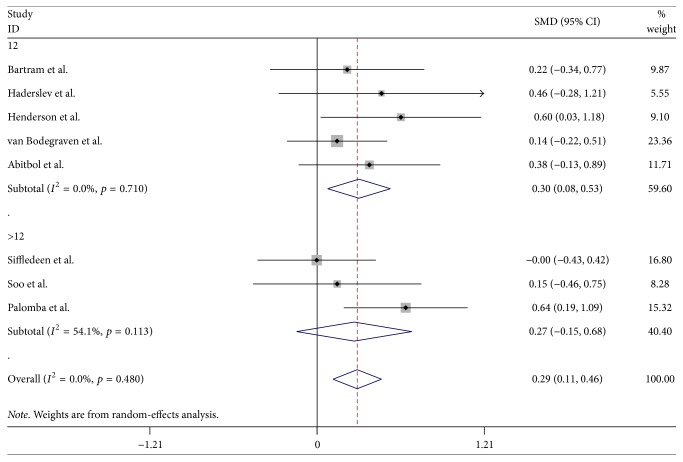
Randomized controlled trials of bisphosphonates in improving total hip BMD in IBD. Forest plot showing the weighted mean differences and 95% confidence interval of the percentage change in bone mineral density at the total hip in the bisphosphonate and control groups. A subgroup analysis of the treatment duration of the two groups is also shown.

Eight studies reported the percentage change in BMD at the total hip in the bisphosphonate groups and control groups [20, 23, 24, 26–30]. The BMD increased at the total hip in the group of IBD patients treated with bisphosphonate (WMD = 0.29, 95% CI: 0.11–0.46, *p* = 0.001) (Figure 4). The test for overall heterogeneity was small (*p* = 0.480). Either funnel plot or Begg's and Egger's tests showed no statistical evidence of publication bias. A further assessment included a subgroup analysis of the treatment duration. Seven trials conducted over 12 months revealed a superior effect in improving BMD of hip (WMD = 0.30, 95% CI: 0.08–0.53, *p* = 0.009). For the other two 24-month trials, the increase in BMD was not significant between the two groups (WMD = 0.27, 95% CI: −0.15–0.68, *p* = 0.206).

### 3.4. Effect of Bisphosphonates in Femoral Neck BMD

The four studies that examined the femoral neck BMD was assessed [20, 27–29]. A summary of the further treatment duration analysis conducted in the related studies is also given. It indicates that the bisphosphonate group was no different from the control group in terms of percentage change in BMD in femoral neck.

### 3.5. New Fractures Analysis

**Figure 5:**
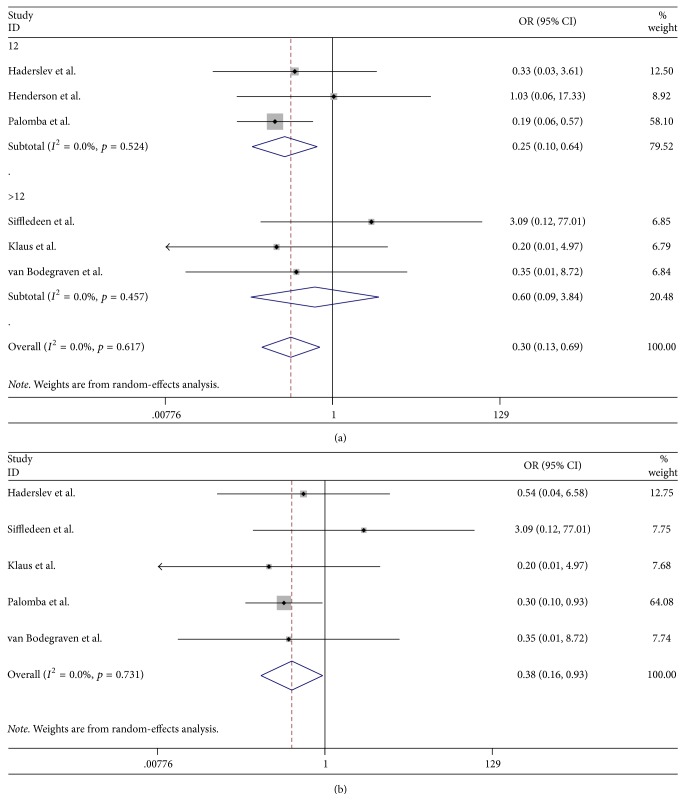
Randomized controlled trials of bisphosphonates in reducing new fractures and vertebrate fractures in IBD. (a) Forest plot showing the odds ratio and 95% confidence interval of the incidence of new fractures of the bisphosphonate and control groups. A subgroup analysis of the treatment duration of the two groups is also shown. (b) Forest plot showing the odds ratio and 95% confidence interval of the incidence of vertebrate fractures of the bisphosphonate and control groups.

Six studies reported the incidences of new fractures during follow-up, including nonvertebral fractures and vertebral fractures [22, 23, 26–28, 30]. The pooled OR of total fractures was 0.30 (95% CI, 0.13–0.69, *p* = 0.005), indicating that bisphosphonate treatment was superior to control treatment in preventing vertebral fractures (OR = 0.38, 95% CI, 0.16–0.93, *p* = 0.035) instead of nonvertebral fractures (OR = 0.35, 95% CI, 0.06–1.95, *p* = 0.228) (Figure 5). Furthermore, pooled data showed significant effectiveness of bisphosphonates compared with controls at 12 months, with the ORs of 12 months and 24 months being 0.25 (95% CI, 0.10–0.64, *p* = 0.004) and 0.60 (95% CI, 0.09–3.84, *p* = 0.587) (Figure 5), respectively.

### 3.6. Adverse Event Analysis

**Figure 6:**
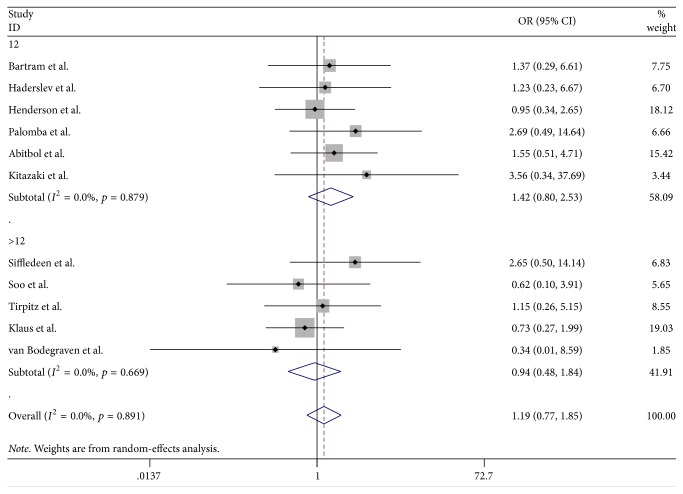
Randomized controlled trials of bisphosphonates in adverse events. Forest plot showing the odds ratio and 95% confidence interval of the adverse event rates.

**Figure 7:**
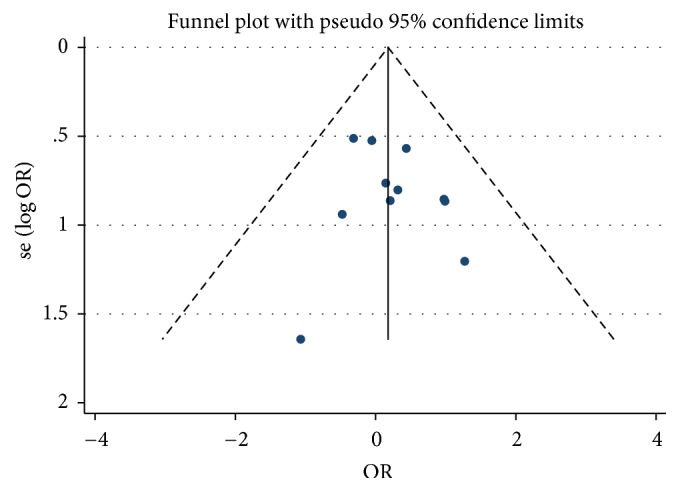
Funnel plot of studies included in Figure 6. Dots represent the results of each study. The funnel plot was visually examined, showing symmetrical characteristic of eleven trials reporting the adverse events. There was no asymmetry and statistical evidence of publication bias among the included studies.

All eleven of the studies showed the number of adverse events. No significant difference was found between the bisphosphonate and control groups (OR = 1.19, 95% CI, 0.77–1.85, *p* = 0.426) (Figure 6), demonstrating that bisphosphonate treatment was not associated with an increased incidence of adverse effects. Incidences of adverse effects were not increased with treatment duration. For 12 months and 24 months, the pooled ORs were 1.42 (95% CI, 0.80–2.53, *p* = 0.227) and 0.94 (95% CI, 0.48–1.84, *p* = 0.848), respectively. The shape of the funnel plot and the Egger's test (*p* = 0.477) (Figure 7) indicated no publication bias. Most of adverse events were gastric and intestinal diseases such as gaseous distention, bloating, and diarrhea. Three trials reported arthralgia [20, 26, 29] and two trials reported reversible bone pain [22, 27].

## 4. Discussion

IBD is a chronic and incurable disease, with increasing numbers of patients suffering from it. Among its complications, osteoporosis and fragility fracture are increasing common, especially in elderly patients. Long-term corticosteroid therapy has been considered one of the major causes of osteoporosis. Medications like bisphosphonates, vitamin D, calcitonin, teriparatide, parathyroid hormone, infliximab, and denosumab are effective for the prevention and treatment of osteoporosis [32]. Bisphosphonates can specifically bind to hydroxyl apatite in bone and inhibit osteoclast activity [14, 33] and is an effective treatment for GC-induced osteoporosis and postmenopausal osteoporosis. The studies enrolled in this meta-analysis showed that bisphosphonates can also reduce the risk of fractures in IBD patients. Only one clinical trial thus far showed that intranasal calcitonin is not able to increase BMD in young IBD patients [34]. Two prospective studies revealed the beneficial effect of infliximab on bone metabolism both in CD and in UC patients [35, 36]. For teriparatide, whether parathyroid hormone and denosumab can improve BMD or reduce the risk of fracture in IBD patients remains unknown.

This meta-analysis and systematic review was conducted to evaluate the effect of bisphosphonates in the prevention and treatment of bone loss in patients with IBD. The incidence of fractures and adverse events was also determined. Eleven RCTs were conducted in the analysis and contributed to some or all of the results of interest. The meta-analysis revealed that there was an increment in BMD at the lumbar spine and total hip in patients treated with bisphosphonates compared with the control group. Although the changes in lumbar spine and total hip BMD showed more significant improvement at 12 months, no difference was found at 24 months. No significant difference was found between the two groups in terms of BMD at the femoral neck, perhaps because the number of documents that noted the changes in the femoral neck BMD was small and the data we selected were taken from different trials. Also, there were six studies reporting the incidences of new fractures during follow-up, five of which reported vertebral fractures and three of which reported nonvertebral fractures, and significant difference between the treatment group and the control group can be seen, showing that bisphosphonates can reduce the risk of fractures, especially vertebral fractures. Similarly, short-term treatment showed significant improvement while long-term treatment showed no difference. There are several explanations for these conflicted results. First, only three trials reported BMD change and the incidence of new fractures in the 24-month duration and therefore may not reflect the difference. Second, a longer duration and a high rate of withdrawal may have affected the results. Bisphosphonates had significant effect on the BMD in the lumbar spine and total hip compared with the placebo and no intervention groups as well as on the prevention of vertebral fractures.

From a bisphosphonate safety perspective, we could not find any statistically significant difference in the occurrence of adverse events between the bisphosphonate and control groups. Most of the adverse events were gastrointestinal reactions to the bisphosphonates in our review. At the same time, IBD itself also contributed to the adverse events. When the participants were aware of the treatment they were receiving, they might have been more or less likely to report adverse events. No other serious adverse events were described in the review. As such, no conclusions can be drawn in relation to the adverse events caused by bisphosphonates for patients with IBD.

We explored the presence of statistical heterogeneity using a chi-squared test and measured the quantity of heterogeneity by *I*^2^. It showed a moderate heterogeneity at the lumbar spine (*I*^2^ = 43.4%, *p* = 0.078). To address our heterogeneity concerns, we used both fixed and random-effects models to make the chosen model more sensitive, and the results were coincident. There are several reasons for this heterogeneity. First, heterogeneity was brought into the meta-analysis by the study we included. In terms of the trials conducted by von Tirpitz et al. [25] and Klaus et al. [22], the number of participants was significantly different between the experiment and control groups when analysing the adverse event ratio. Postmenopausal women were excluded from Abitbol's [20] trial and exclusively included in Palomba's [26] trial suggesting that the result of this study should interpreted with caution. Second, the data related to the percentage change in BMD was limited because some citations only gave baseline and after-treatment *T*-scores [25] and the percentage change in the *T*-scores [22]. The primary statistics could not be found. In the femoral neck BMD, only four RCTs [20, 27–29] were reported. Third, our meta-analysis was based on published data; unpublished data was excluded, and heterogeneity was found. Finally, different bisphosphonate doses and administration regimen might have been potential sources of the observed heterogeneity.

Our meta-analysis was limited in several ways. All eleven of the included trials had small sample sizes, with an average of 78 participants with IBD. Small trials have less power, meaning that there was a lower chance of detecting a small but true effect as statistically significant. Another limitation was that seven RCTs focusing on Crohn's disease [22–25, 27, 29, 30] and three RCTs focusing on IBD were accepted [20, 26, 28] and one trial focusing on ulcerative colitis was accepted [31]. The number of trials focusing on IBD was relatively small. One trial assessed pamidronate versus no intervention, two trials assessed alendronate versus placebo and no intervention, respectively, two trials assessed ibandronate versus no intervention, one trial assessed etidronate versus no intervention, four trials assessed risedronate versus placebo, and one trial assessed clodronate versus placebo.

In summary, our integration of the available individual clinical trials indicated that some advantages were observed between the bisphosphonate and control treatments for BMD in the lumbar spine and total hip regions of patients with IBD. The bisphosphonates were found to be effective and safe. Clinicians should consider these results when choosing treatments for IBD patient with bone loss.

## Figures and Tables

**Table 1:** Summary of the basic characteristics of the bisphosphonate and control groups.

Trial	Year	Intervention	Bisphosphonate administration	Control	Number of patients (intervention/ control)	Age (intervention/ control)	BMD (±SD g/cm^2^) lumbar spine (intervention/ control)	BMD (±SD g/cm^2^) Hip (intervention/ control)	BMD (±SD g/cm^2^) *T*-score lumbar spine (intervention/ control)	BMD (±SD g/cm^2^) *T*-score lumbar spine (intervention/ control)	Duration
Bartram et al.	2003	Pamidronate	30 mg/3 months i.v	No placebo	37/37	45.1 ± 11.4/ 43.5 ± 12.3	0.87 ± 0.09/0.86 ± 0.08	0.73 ± 0.01/0.78 ± 0.09	−1.84 ± 0.82/−1.91 ± 0.72	−2.34 ± 0.79/−1.92 ± 0.77	12
Haderslev et al.	2000	Alendronate	10 mg/day p.o	Placebo	15/17	44 ± 13/44 ± 12	0.74 ± 0.10/0.77 ± 0.09	0.74 ± 0.10/0.77 ± 0.09	−1.53/−1.21	−1.50/−1.70	12
Siffledeen et al.	2005	Etidronate	400 mg/14 days p.o	No placebo	71/72	40.0 ± 12.1/40.1 ± 14.1	0.94 ± 0.10/0.91 ± 0.11	0.86 ± 0.11/0.85 ± 0.11	−1.30 ± 0.85/−1.04 ± 0.75	−0.99 ± 0.72/−1.57 ± 0.97	24
Soo et al.	2012	risedronate	35 mg/day p.o	Placebo	45/43	39.8 ± 13.7/36.7 ± 12.7	0.936 ± 0.095/0.899 ± 0.075	0.886 ± 0.080/0.898 ± 0.097	−1.2 ± 0.9/−1.5 ± 0.7	−0.7 ± 0.6/−0.7 ± 0.6	24
Tirpitz et al.	2003	Ibandronate	1 mg/3 months i.v	No placebo	35/13	35.7 ± 1.8/37.15 ± 3.0	NA	NA	−2.29 ± 0.11/−1.57 ± 0.1	−2.29 ± 0.11/−1.57 ± 0.1	27
Klaus et al.	2011	Ibandronate	1 mg/3 months i.v	No placebo	54/32	33.8 ± 9.76/36.8 ± 13.1	0.90 ± 0.04/0.85 ± 0.08	0.90 ± 0.04/0.85 ± 0.08	−1.57 ± 0.31/−1.89 ± 0.71	−1.57 ± 0.31/−1.89 ± 0.71	42
Henderson et al.	2006	risedronate	5 mg/day p.o	Placebo	30/31	49.9 ± 12.8/47.2 ± 11.4	0.908 ± 0.082/0.898 ± 0.116	0.899 ± 0.127/0.867 ± 0.01	NA	NA	12
Palomba et al.	2005	risedronate	35 mg/week p.o	Placebo	45/45	52.3 ± 3.2/51.4 ± 3.0	0.561 ± 0.063/0.540 ± 0.062	0.561 ± 0.06/0.540 ± 0.062	NA	NA	12
van Bodegraven et al.	2014	risedronate	35 mg/week p.o	Placebo	64/67	43 ± 13/42 ± 13	0.94 ± 0.11/0.95 ± 0.11	0.81 ± 0.09/0.81 ± 0.11	−1.30 ± 0.61/−1.26 ± 0.78	−1.22 ± 0.63/−1.21 ± 0.51	24
Abitbol et al.	2007	Clodronate	900 mg/3 months i.v	Placebo	33/34	30/30	NA	NA	NA	NA	12
Kitazaki et al.	2009	alendronate	5 mg/day p.o	No placebo	19/20	41.2 ± 12.8/38.1 ± 15.5	0.926 ± 0.098/0.906 ± 0.125	NA	NA	NA	12

**Table 2:** Summary of the number of adverse events, nonvertebral and vertebral fractures between the bisphosphonate and control groups.

Trial	Year	Bisphosphonate group				Control group			
		Number of patients	Number of adverse events	Number of nonvertebral fractures	Number of vertebral fractures	Number of patients	Number of adverse events	Number of nonvertebral fractures	Number of vertebral fractures
Bartram et al.	2003	37	4			37	3		
Haderslev et al.	2000	15	12	0	1	17	13	1	2
Siffledeen et al.	2005	71	5		1	72	2		0
Soo et al.	2012	45	2			43	3		
Tirpitz et al.	2003	35	9			13	3		
Klaus et al.	2011	54	12		0	32	9		1
Henderson et al.	2006	30	18	1		31	19	1	
Palomba et al.	2005	45	5	0	5	45	2	4	14
van Bodegraven et al.	2014	64	0		0	67	1		1
Abitbol et al.	2007	33	26			34	24		
Kitazaki et al.	2009	19	3			20	1		
